# Fluorescence/luminescence-based markers for the assessment of *Schistosoma mansoni* schistosomula drug assays

**DOI:** 10.1186/s13071-015-1233-3

**Published:** 2015-12-08

**Authors:** Gordana Panic, Dayana Flores, Katrin Ingram-Sieber, Jennifer Keiser

**Affiliations:** Department of Medical Parasitology and Infection Biology, Swiss Tropical and Public Health Institute, CH–4002 Basel, Switzerland; University of Basel, CH–4003 Basel, Switzerland

**Keywords:** *Schistosoma mansoni*, Newly Transformed Schistosomula, Drug sensitivity assay, Fluorescence, Luminescence, Viability marker, Cytotoxicity marker

## Abstract

**Background:**

Schistosomiasis is responsible for a tremendous public health burden, yet only a single drug, praziquantel, is available. New antischistosomal treatments should therefore be developed. The accuracy, speed and objectivity of *in vitro* drug screening depend on the assay read-out. Microscopy is still the current gold standard and is in need of updating to an automated format. The aim of the present study was to investigate a panel of fluorescence/luminescence dyes for their applicability as viability markers in drug sensitivity assays for *Schistosoma mansoni* schistosomula.

**Methods:**

A search for available viability and cytotoxicity marker assays and dyes was carried out and a short-list of the most interesting candidates was created. The selected kits and dyes were tested on *S. mansoni* Newly Transformed Schistosomula (NTS), first to assess whether they correlate with parasite viability, with comparatively low background noise, and to optimise assay conditions. Markers fulfilling these criteria were then tested in a dose–response drug assay using standard and experimental drugs and those for which an IC_50_ value could be accurately and reproducibly calculated were also tested on a subset of a compound library to determine their hit-identification accuracy.

**Results:**

Of the 11 markers selected for testing, resazurin, Vybrant® and CellTiter-Glo® correlated best with NTS viability, produced signals ≥ 3-fold stronger than background noise and revealed a significant signal-to-NTS concentration relationship. Of these, CellTiter-Glo® could be used to accurately determine IC_50_ values for antischistosomals. Use of CellTiter-Glo® in a compound subset screen identified 100 % of hits that were identified using standard microscopic evaluation.

**Conclusion:**

This study presents a comprehensive overview of the utility of colorimetric markers in drug screening. Our study demonstrates that it is difficult to develop a simple, cheap “just add” colorimetric marker-based drug assay for the larval stage of *S. mansoni.* CellTiter-Glo® can likely be used for endpoint go/no go screens and potentially for drug dose–response studies.

**Electronic supplementary material:**

The online version of this article (doi:10.1186/s13071-015-1233-3) contains supplementary material, which is available to authorized users.

## Background

Schistosomiasis, causing 3.3 million DALYs lost, is one of the most important Neglected Tropical Diseases (NTDs) [1]. It is a water-borne trematodiasis caused by any of six *Schistosoma* species that parasitise humans: *Schistosoma haematobium, S. mansoni, S. japonicum, S. mekongi, S. guineensis* and *S. intercalatum*- the former 3 being most common [2, 3]. Schistosomiasis is prevalent mainly in rural areas of poor sanitation, with the majority of cases occurring in Sub-Saharan Africa [3, 4]. The bottom line of the WHO schistosomiasis control programme is morbidity reduction via preventative chemotherapy [3]. Only a single drug- praziquantel- is used to treat millions of people annually and its coverage is projected to reach 235 million people by 2018, which raises concerns of increasing drug pressure [5, 6]. Moreover, although praziquantel is a safe and cheap therapy effective on the adult stage of the disease, it is inactive against the juvenile stage [6, 7]. Therefore, new chemotherapies are desperately needed.

In the past, standard operating procedures (e.g. established at TDR-designated compound screening centers) relied on adult worms incubated with the candidate drugs for 72 hours, after which their viability is assessed microscopically [8]. This method requires the intensive use of mice (as no *in vitro* life cycles currently exist), is time consuming and low-throughput. Thus recently, the use of Newly Transformed Schistosomula (NTS) has been popularised as a higher-throughput screen [9–12]. Nonetheless, assessing parasite viability via microscopic read-out is slow and subjective and is therefore a bottle-neck for high-throughput screening. Consequently, with recent advancements in automated technologies, a number of assay read-out alternatives have been attempted with varying degrees of success [9]. These methods include the measurement of parasite mobility over time via electrical impedence with xCELLigence [13], isothermal microcalorimetry [14] and automated image-based Bayesian classification [15]. On the other hand, using dye-based assays that can be read by an automatic plate reader would be a simpler, cheaper, more practical and more trainable read-out alternative, requiring little extra equipment or software. Three fluorescent and one luminescent assay reagent have been studied on *S. mansoni* NTS to date: the Alamar Blue® viability assay (resazurin), the fluorescein diacetate/ propidium iodide fluorescent multiplex assay and a fluorometric L-lactate assay (fluorescent) and CellTiter-Glo® (luciferase) as the luminescent assay. In more detail, Alamar Blue® could discriminate between live and dead NTS after 7 days of incubation with standard drugs but not for earlier time-points and it could not be used to measure dose–response drug effects [11]. The fluorescein diacetate/ propidium iodide assay is a duplex viability and cytoxicity assay where fluorescein diacetate stains live NTS and propidium iodide stains dead NTS [16]. This assay was successful in that it could be used to calculate an LD_50_ value for auranofin, an antirheumatic agent that is active against *S. mansoni,* and could distinguish between dead and alive NTS for several standard drugs. However, practical issues, such as the requirement of a rinsing step and the need for a high number of NTS, as well as its questionable ability to determine dose-dependent effects for all standard drugs, were elements that could be improved upon. Howe and colleagues investigated lactate, a byproduct of glycolysis known to be secreted via aquaglyceroporins from NTS and adult worms, as a possible marker for viability. They too were able to generate dose–response curves for some but not all standard drugs using a commercial L-lactate kit [17]. Nonetheless, the procedure requires removing the supernatant from the drug assay (without aspirating the NTS) and then diluting it to an acceptable fluorescence range as needed, rendering it less than high-throughput. More recently, Lalli and colleagues (2015) validated the use of the commercial luminescence-based cell viability kit, CellTiter-Glo®, in an *in vitro* assay using *S. mansoni* NTS and adult worms [18]. Here, however, a precise multi-drop dispenser was required to ensure an exact number of NTS was present in each well. Hence, although the investigation of marker-dye based assays has been a popular pursuit, the aim of a simple, inexpensive and precise dye that does not require much additional equipment or analysis has not entirely been met.

In the present study we sought to identify an easy-to-use, “just-add” viability or cytotoxicity marker assay that can accurately determine the viability of NTS in a drug sensitivity screen. We therefore reviewed the literature for previous use of dyes and markers on *S. mansoni* for their potential use as viability or cytotoxicity markers in an automated drug assay. We also researched the market for commercially available viability and cytotoxicity kits and dyes that could in theory be adapted for use in an *S. mansoni* NTS drug assay. Eleven markers, resazurin (the active component of Alamar Blue®), OmniCathepsin™, CellTiter-Glo®, Vybrant®, CytoTox- ONE™, LIVE/DEAD® viability/cytotoxicity kit-, ApoTox-Glo™, CellTox™ Green Cytotoxicity Assay, DAPI, Hoechst 33258 (bis-Benzimide) and FluoForte® Calcium Assay with diverse modes of action were selected. All markers were tested to elucidate whether wells containing NTS could produce signals significantly stronger than wells containing medium only, a significant signal-to-NTS concentration relationship and if differential signals between live and dead NTS could be observed. For the three markers resazurin, Vybrant® and CellTiter-Glo® meeting this criteria, dose–response drug assays were conducted. CellTiter-Glo® was further validated on a 25-compound subset of a compound library of FDA-approved drugs.

## Methods

### Literature search

A recent review on approaches to measuring helminth viability [9] served as a starting point to investigate dyes and methods that had already been attempted to measure *S. mansoni* viability. Each marker listed in the publication was checked to see how it was used on *S. mansoni*. Specifically, we looked if the named marker had already been used on NTS in a multi-well assay. In parallel, a simple Google and PubMed search was conducted in order to identify commercial viability/cytotoxicity kits available on the market or markers and dyes that are not normally used to measure viability but could be used to measure *S. mansoni* viability in theory. Terms used were “colorimetric viability markers”, “colorimetric viability assays”, “viability assays”, “fluorescent viability markers”, “cytotoxicity assays”, “cytotoxicity marker assays”, “*Schistosoma mansoni* cytotoxicity”, “*Schistosoma mansoni* viability” and “*Schistosoma mansoni* assay”. With the aim of identifying desirable candidate markers for NTS, the following primary exclusion criteria were applied: (i) active component should not be one that has already been identified as ineffective at measuring viability in NTS (based on published or unpublished data); (ii) must not rely on cellular replication since NTS do not replicate or grow very fast; (iii) must not require additional rinsing steps since NTS do not fix to the bottom of well plates. In addition, it was desirable that the marker did not also measure bacteria and fungi viability and would not be too costly.

### Media, chemicals and drugs

Medium 199 was purchased from Gibco (Basel, Switzerland), inactivated foetal calf serum (iFCS) was obtained from Connectorate AG (Dietikon, Switzerland) and a mixture of penicillin-streptomycin (10,000 units/ml penicillin and 10 mg/ml streptomycin) was purchased from Sigma-Aldrich (Buchs, Switzerland).

CellTiter-Glo®, ApoTox-Glo™, CellTox™ Green Cytotoxicity Assay and CytoTox-ONE™ were purchased from Promega. The Vybrant® Cytotoxicity Assay Kit and the LIVE/DEAD® Viability/Cytotoxicity Kit for mammalian cells were purchased from Molecular Probes (Invitrogen), whereas FluoForte® Calcium Assay kit was acquired from Enzo Life Sciences Inc. DAPI (4’, 6-Diamidino-2-phenylindole dihydrochloride) and Hoechst 33258 were purchased from Sigma-Aldrich and stock solutions of 5 mg/ml were constituted by dissolving the substrate in dH_2_O and DMSO respectively. The OmniCathepsin™ reagent was prepared by dissolving OmniCathepsin™ substrate (Enzo Life Sciences) in DMSO solution at a concentration of 10 mM. The resazurin dye was constituted by dissolving resazurin sodium salt (Sigma) in 1x PBS solution at a concentration of 125 mg/l. All markers were stored at –20 °C until use except DAPI and Hoechst 33258, which were stored at 4 °C.

Praziquantel, and mefloquine were purchased from Sigma-Aldrich (Buchs, Switzerland), and oxamniquine was donated by Dr. Quentin Bickle. Drug stock solutions were made by dissolving the compounds in DMSO (dimethyl sulfoxide, Fluka, Buchs, Switzerland) at a concentration of 10 mM and were stored at −20 °C until use.

### Transformation of S. mansoni cercariae into Newly Transformed Schistosomula (NTS)

For the transformation of cercariae into schistosomula, a cercarial suspension was collected from *S. mansoni-*infected *Biomphalaria glabrata* snails and subjected to a mechanical *in vitro* transformation described previously [12]. The NTS were then placed in warm culture medium: Medium 199 supplemented with 5 % iFCS and 1 % penicillin-streptomycin mixture and incubated for 24 hours at 37° C and 5 % CO_2_ until use.

### Signal correlation to NTS concentration assays and exposure time assays

Assays were set up to measure whether incubation with the selected marker yielded a significant 3:1 signal-to-background (S/B) ratio. In addition, viability markers should present a linear curve between the signal (fluorescence or luminescence) and increasing concentrations of live NTS, but a poor signal and no relationship when incubated with dead NTS and vice-versa when cytotoxicity markers are used. Thus for each marker, an assay with increasing concentrations of NTS/well (20, 40, 60, 80, 100, 200, 300 and 400 NTS/well), of live and dead NTS was set up. To measure signal strength in correlation to exposure time to the marker, the NTS concentration assays were measured at multiple time-points (Additional file 1: Table S1). In addition, because it was noted for Cell-Tox™ Green that it mattered for signal intensity how NTS were killed, a variety of substances were tested to kill the NTS for the potential cytotoxicity markers, the FluoForte® Calcium Assay, DAPI and Hoechst 33258 (Additional file 1: Table S1).

The total well volume, including the marker, was between 100 and 250 μl. Control wells contained culture medium only (supplemented Medium 199). Altogether, 2 to 3 trials of duplicates/triplicates were performed for each marker. Marker-specific methods (the amount of marker added, the plate incubation times and the excitation/emission (Ex/Em)) are summarised in Additional file 1: Table S1. Spectra were determined by the commercially published protocols or in the case of resazurin, Hoechst 33258 and DAPI, from previous publications [11, 19–21]. Luminescence or fluorescence was read using the SpectraMax® M2 Multi-Mode Microplate Reader (Molecular Devices).

For OmniCathepsin™, preliminary assays were done to determine the optimal concentration of the dye. LIVE/DEAD® and ApoTox-Glo™ are duplex assays, and thus the plates were scanned twice, each time at the Ex/Em spectra specified for each dye (Additional file 1: Table S1).

Since LIVE/DEAD®, CellTox™ Green Cytotoxicity, DAPI and Hoechst 33258 are fluorescence markers that stain components within the NTS themselves, read-outs were complemented by confirmation of NTS staining via fluorescence microscopy. From the assay, 20 μl of live and dead NTS suspensions were placed on glass slides with cover slips. Inspection was conducted using the Leica DM5000B upright microscope. The L5 filter was used to view objects stained by calcein from the LIVE/DEAD® kit and CellTox™ Green Cytotoxicity Assay cyanine dye; the Texas Red (TR) filter was used to view objects stained by EthD-1. The CY3 filter was used for DAPI and the A4 filter for Hoechst 33258. Imaging was possible via the microscope camera, which was connected to Leica Application Suite 2.4.0 imaging software.

### Drug sensitivity assay

Markers that showed an S/B of 3:1 and a strong signal-to-NTS concentration relationship were selected for testing with two standard and one experimental drug to assess if they could be used to determine IC_50_ values in a dose–response drug sensitivity assay. Drugs were serially diluted to fit a range of previously reported IC_50_ values [22, 23]. The dilution series were as follows: 20, 10, 5, 2.5, 1.3 and 0.7 μM for praziquantel and mefloquine; and 240, 120, 60, 30, 15, 7.5 and 3.7 μM for oxamniquine.

The drug assays were set up according to manufacturer protocols using 200 NTS and an incubation time of 24 hours with resazurin, 15 min with Cell-Titer-Glo® and 70 min with Vybrant®. Assays were evaluated at various time-points. Each SpectraMax® read-out was accompanied by a microscopic read-out in order to compare the two methods. Microscopic assessment of viability was done by observing the NTS morphology and motility and by assigning the NTS viability scores as follows: 0 = dead; 1 = both slower movement and damage to tegument or severely impaired movement or severely damaged tegument; 2 = slow movement or notable damage to tegument; 3 = lively movement and undamaged tegument. IC_50_ values, which describe the drug concentration at which worm viability is inhibited by 50 % as scored by the viability scare, were calculated as described below using both read-outs and compared.

### Assay validation

To confirm that CellTiter-Glo® could be reliably used in a drug screen, a subset (*n* = 25) of a previously screened library of FDA approved compounds [24] was chosen for re-screening with CellTiter-Glo®. Compounds were selected by randomly picking from the “hit” and “not hit” lists, such that the hit rate in the assay would be ~20-25 %. The compounds were screened on NTS at 10 μM and CellTiter-Glo® was added as previously described. Each compound was tested in duplicate twice, along with an NTS-free blank for background measurement. Assays were assessed by both a microscopic evaluation and the SpectraMax® scan. Hit compounds were defined as those compounds that achieved ≥ 75 % reduction in viability (calculated using the viability score described above) for the microscopic evaluation and ≥ 75 % reduction in fluorescence signal relative to live controls. Dead controls were included in the assay to calculate the Z’ factor for each plate.

### Statistical analysis

Averages, standard deviations and S/B ratios were calculated and graphs generated with Microsoft Office Excel 2013. Dose–response drug sensitivity assays using the markers were read both by the SpectraMax® and manually via microscopic read-out. From the SpectraMax® read-outs, the IC_50_ values were calculated in SoftMax® Pro. From the microscopic read-out, the IC_50_ values were calculated with the help of CompuSyn® (2006). The Z’ factor was calculated according to the formula described by Zhang *et al.,* where a score ≥ 0.5 is considered excellent [25].

## Results

### Literature search

A substantial number of dyes have been tested on the different stages of *S. mansoni* and many of them specifically on the NTS stage (Additional file 2: Table S2). Many dyes, however, were not suitable for automated drug sensitivity assay read-outs, the reasons for which are presented in Additional file 2. Our search identified 2 viability, 3 cytotoxicity, 2 multiplex and 4 “experimental” markers for further testing. Their features/mechanism of action are summarised in Additional file 2: Table S2.

### Signal to NTS concentration assays and exposure time assays

#### Viability markers

For all assays, optimal incubation parameters and key results are summarised in Table 1.

**Table 1:** Summary of marker optimizations and results

Marker Type	Marker Assay	Optimal incubation time	S:B ratio ≥ 3:1?	Correlation to NTS viability/cytotoxicity	Selected for drug assay testing?	Justification
Viability Markers	CellTiter-Glo®	15 min	Yes, with ≥ 100 NTS	Strong	Yes	Met criteria
	Resazurin	24 hours	Yes, with ≥ 200 NTS	Strong	Yes	Met criteria
Cytotoxicity Markers	Vybrant®	70 min	Almost, with ≥ 300 NTS	Strong up to 300 NTS	Yes	Met criteria
	CytoTox-ONE	2 hours	No	Strong	No	S:B ratio low, large standard deviations between data points
	CellTox™ Green Cytotoxicity Assay	24 hours	Yes, with ≥ 400 NTS	Strong with ≥ 400 NTS	No	Viable NTS died within 24 hours of exposure to dye; strong signals from completely lysed cells only
Multiplex Markers	LIVE/DEAD® Viability/Cytotoxicity Kit	Does not exist	No	Poor for both live and dead NTS	No	Poor correlation to NTS viability/cytotoxicity
	ApoTox-Glo™	Does not exist	No	Strong for live-cell marker, poor for dead-cell marker	No	Markers induced spazzing and death of NTS after 6 hours
Experimental Markers	OmniCathepsin™	2 hours (with 10 μM marker concentration)	No	Good: Differential signals for live vs. dead observed	No	S:B ratio too low, not enough difference between live and dead NTS
	FluoForte® Calcium Assay	1.5 hours with ≥ 200 dead NTS	Yes, with ≥ 200 dead NTS (lysed only)	Poor: strong staining for completely lysed cells only	No	Stains only completely lysed cells
	DAPI	15 min (with 10 μg/ml dye concentration)	No	Good: stained many dead NTS phenotypes	No	Background fluorescence too high, signals too low
	Hoechst 33258	15 min (with 1 μg/ml dye concentration)	No	Good: stained many dead NTS phenotypes	No	Background fluorescence too high, signals too low

**Fig. 1:**
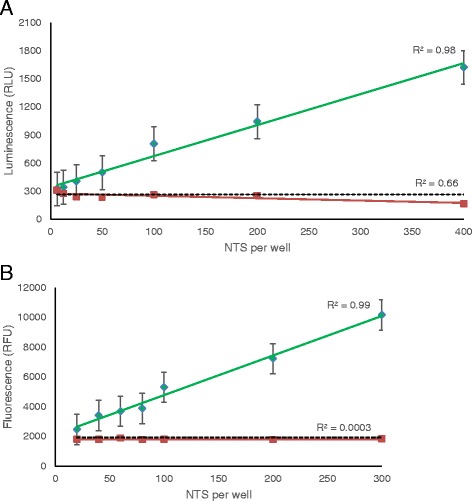
Correlation to NTS concentration for viability markers **a** CellTiter-Glo® and **b** resazurin. Green lines represent data from live NTS and red lines represent data from dead NTS. The dotted line represents the background signal from medium-only wells. Curves are shown for the optimal incubation time for each marker: 15 min for Cell-Titer Glo and 24 hours for resazurin.

CellTiter-Glo® assay results showed a strong correlation between live NTS number and luminescence signal (R^2^ = 0.98) (Fig. 1a). The signals were strongest at 15 min incubation time with the reagent, after which the signal strength decreased. A S/B ratio of at least 3:1 was observed from 100 or more NTS/well. Dead NTS did yield a luminescence signal but comparable to that of the background luminescence.

Resazurin presented a strong linear relationship with live NTS concentration (R^2^ = 0.98) but revealed large standard deviations (Fig. 1b). The signals, S/B ratio and R-squared values were highest after 24 hours of incubation. The minimum number of live NTS that gave at least a 3:1 S/B ratio was 200 NTS. The fluorescence signal for dead NTS was low (183.6 ± 40.3 RFU at 300 dead NTS when subtracted from background) and there was not a strong correlation between number of dead NTS and the fluorescence signal (R^2^ = 0.003).

### Cytotoxicity markers

**Fig. 2:**
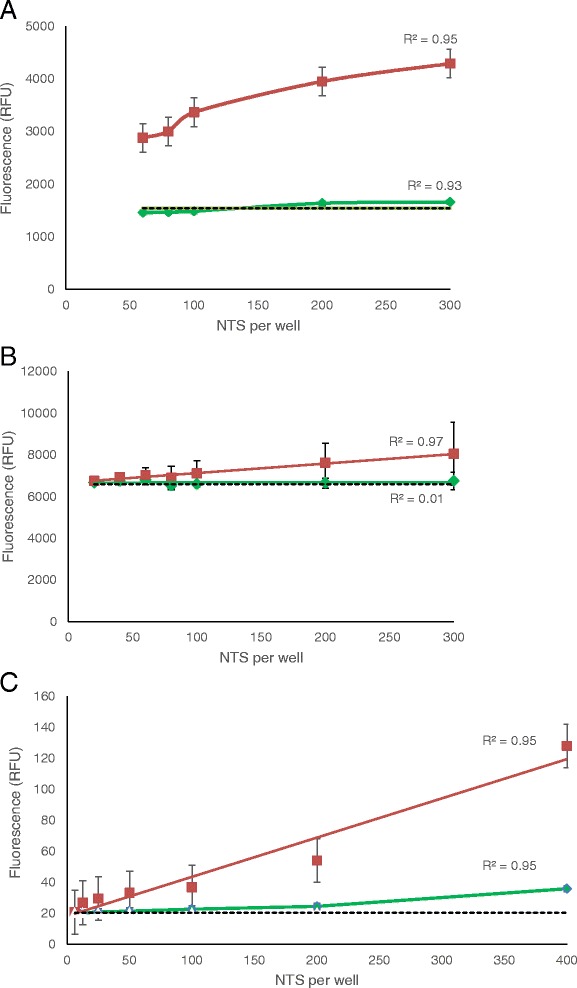
Correlation to NTS concentration for cytotoxicity markers **a** Vybrant®, **b** CytoTox-ONE™ and **c** CellTox™ Green. Green lines represent data from live NTS and red lines represent data from dead NTS. The dotted line represents the background signal from medium-only wells. Curves are shown for the optimal incubation time for each marker: 70 min for Vybrant®, 2 hours for CytoTox-ONE and 24 hours for CellTox™ Green.

Dead NTS incubated with Vybrant® yielded a strong signal-to-dead-NTS concentration correlation up to 300 dead NTS (R^2^ = 0.95), after which the signal plateaued. The highest S/B ratio was obtained after 70 min with 300 dead NTS per well (S/B = 2.78:1), though this fell just below the 3:1 cut-off. The signals for live NTS were comparable to background levels (Fig. 2a).

A strong correlation between dead NTS concentration and fluorescence signal (R^2^ = 0.97) was observed for CytoTox-ONE™, however, with very large standard deviations for each data point. Live NTS signals were comparable to background levels but background levels for CytoTox-ONE™ were very large (6605 RFU) (Fig. 2b). As a result, the S/B ratio was always at around 1:1 regardless of dead NTS concentration or exposure time to CytoTox-ONE™.

CellTox™ Green showed a linear relationship between fluorescence and number of dead NTS per well, exhibiting a significant signal at a concentration from 400 NTS and onwards at 24 hours (Fig. 2c). Fluorescence microscopy after 4 hours incubation with the marker showed that the dye was effectively penetrating the cell Additional file 3: Figure S1(E, F), though the signal intensity and relationship linearity measures are significant from 24 hours after addition of reagent (S/B >3:1). Additionally, live NTS measured by this reagent gave low fluorescence signals (S/B <2:1). However, after 24 hours of exposure to CellTox™ Green, live NTS viability was severely diminished and hence could not be used as reliable controls at this time-point measurement. An important point is that only lysed NTS, and not heat, EtOH 10 %, DMSO 25 %, praziquantel nor mefloquine – killed NTS, showed significant values compared with the control wells.

### Multiplex assays

**Fig. 3:**
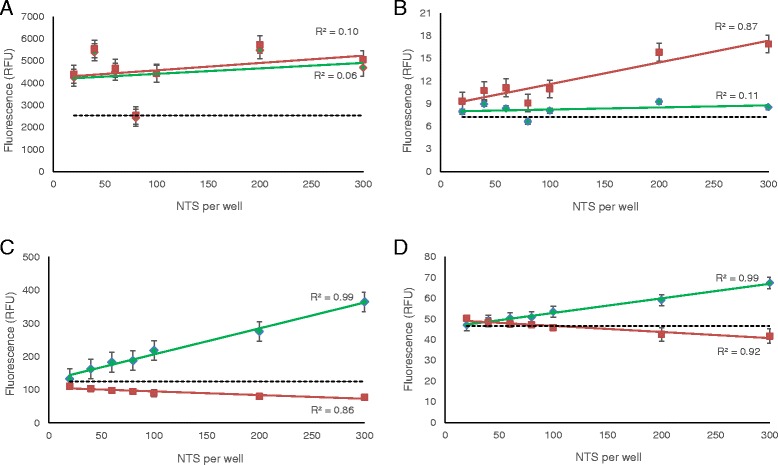
Correlation to NTS concentration for multiplex markers **a** and **b** LIVE/DEAD® Viability/Cytotoxicity Kit and **c** and **d** ApoTox-Glo™. Since these kits contained both a viability and cytotoxicity reagent, a separate scan was done for the viability reagent (**a**) for LIVE/DEAD® and (**c**) for ApoTox-Glo™ and for the cytotoxicity agent (**b**) for LIVE/DEAD® and (**d**) for ApoTox-Glo™. The green curve is for live NTS, the red is for dead NTS and the dotted line is for background signals. The graphs presented correspond to the optimal incubation time for each marker, which is 24 hours for both LIVE/DEAD® and ApoTox-Glo™.

Using the LIVE/DEAD® Viability/Cytotoxicity Kit, measurements with calcein showed weak signals and a poor correlation with live NTS concentration (Fig. 3a). However, microscopic images demonstrate that at 45 minutes after exposure to the dyes, the NTS are stained properly and discriminately (Additional file 3: Figure S1(A-D)). Additionally, signals from dead control NTS were very close to the range of the live NTS signals. Meanwhile, signals generated from dead NTS scanned by the EthD-1 Ex/Em spectra yielded an extremely low signal and only a moderate linear correlation (Fig. 3b). The negative controls, however, also showed poor signals and linearity. Measurements with calcein using as many as 1000 NTS showed an improved signal correlation with NTS numbers (R^2^ = 0.89 at 4 hours incubation time) but nonetheless a low fluorescence signal overall (Additional file 4: Figure S2(A)). Measurements with EthD-1 showed no significant improvement in signal and the signal even plateaued at higher NTS concentrations (Additional file 4: Figure S2(B)). The S/B ratio was less than 3 for both calcein and EthD-1 at all time-points.

ApoTox-Glo™ also presented as a poor multiplex assay. The AFC reagent yielded a strong linear relationship with live NTS concentrations and high signal levels (Fig. 3c) but only after 6 hours of exposure to the marker, when microscopic evaluation revealed spastic NTS. By 24 hours, all NTS were dead. The bis-AAF-R110 reagent yielded a very poor signal and NTS concentration correlation with dead NTS (Fig. 3d). Paradoxically, the signal correlation to increasing live NTS concentrations was strong (R^2^ = 0.99).

### Experimental reagents

As the experimental dyes selected for this work have not yet been used as viability or cytotoxicity markers, no standard protocol for reagent concentration or suggested reagent exposure time existed. Hence, initial experiments were conducted to determine these parameters, summarised in Table 1. Thereafter, experiments were conducted to determine if the reagent could produce differential signals for live versus dead NTS.

**Fig. 4:**
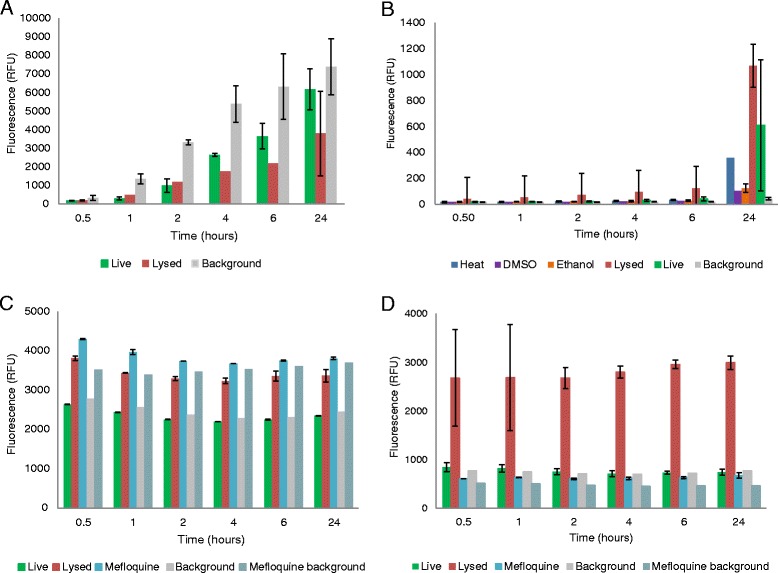
Signals of live and dead NTS over time for **a** OmniCathepsin™, **b** FluoForte® Calcium Assay, **c** DAPI and **d** Hoechst 33258

Using the OmniCathepsin™ assay, high fluorescence signals were yielded by live NTS (Fig. 4a). The signals for live NTS were notably higher than for dead NTS, however, high background signals interfered with the assay, and neither significant differences between live and dead NTS signals, nor a 3:1 S/B ratio could be achieved.

Incubation of dead NTS with FluoForte® Calcium Assay produced significant signal intensity in comparison with live NTS (Fig. 4b). However, this was only the case for completely lysed NTS. NTS killed by heat, EtOH 10 % or DMSO 25 % did not produce high signals. Since no drug completely lyses NTS, further investigations with this assay were discontinued.

For both DAPI and Hoechst 33258, the S/B ratio did not reach the 3:1 threshold, though a difference was observed between the fluorescence values of live versus lysed or mefloquine-killed NTS (Figs 4c and d). When observed with fluorescence microscopy, both dyes stained dead NTS at their determined optimal incubation times and did not stain control live NTS (Additional file 3: Figure S1(E-J)).

Since high background signals were a notable problem for many of these stains, assays with Omnicathepsin™, DAPI and Hoechst 33258 were set up in order to assess the effect of phenol red, iFCS and medium type on the background fluorescence values (Additional file 5: Figure S3). Indeed, both iFCS and phenol red contribute to high background noise though most of it is attributable to presence of iFCS. However, it is not possible for NTS to remain viable without iFCS, hence removing it from the medium or decreasing the concentration are not viable options for a 72 hour drug assay.

### Drug sensitivity assays

Resazurin, Cell-Titer Glo®, and Vybrant® were selected for further testing in a drug sensitivity assay using standard drugs. An automatic read-out with resazurin did not produce dose-dependent fluorescence values and thus IC_50_ values could not be calculated (Table 2). Vybrant® did not yield IC_50_ values for praziquantel and oxamniquine and gave an IC_50_ for mefloquine that was higher than microscopically derived values. In the case of CellTiter-Glo®, an automatic read-out did not produce sufficient dose-dependent values to calculate an IC_50_ for praziquantel. The IC_50_ calculated for oxamniquine was higher than the microscopically determined one, though oxamniquine IC_50_ values fluctuated widely even between microscopic read-outs. Nonetheless, the IC_50_ value obtained for mefloquine was consistent with the microscopic read-out. Furthermore, a single concentration (10 μM) drug screen with CellTiter-Glo® revealed that of the 25 compounds screened, 100 % of positives were identified as such, and 100 % of negatives were also identified as such (Table 3). The plate Z’ scores were ≥ 0.6.

**Table 2:** IC_50_ values generated by resazurin, CellTiter-Glo® and Vybrant® compared to microscopic values

	Resazurin		CellTiter-Glo®		Vybrant®	
Drug	IC_50_ SpectraMax (μM)	IC_50_ Microscopic Readout (μM)	IC_50_ SpectraMax (μM)	IC_50_ Microscopic Readout (μM)	IC_50_ SpectraMax (μM)	IC_50_ Microscopic Readout (μM)
Praziquantel	not calculable	1.7 ± 1.5	not calculable	1.3 ± 0.9	not calculable	2.4 ± 1.2
Mefloquine	not calculable	2.6 ± 1.1	4.9 ± 2.9	2.4 ± 1.3	7.9 ± 1.7	1.7 ± 0.3
Oxamniquine	not calculable	135.1 ± 128.8	194.5 ± 52.6	87.7 ± 49.7	not calculable	20.3 ± 9.5

**Table 3:** CellTiter-Glo® and microscopic evaluation of a small subset (*n* = 25) of an FDA-approved compound library. Values of % reduced viability of compound-treated NTS relative to the controls were based on average luminescence for CellTiter-Glo® (subtracted from background) and on average viability scores for the microscopic evaluation.

	% reduced viability relative to controls	
Compound	CellTiter-Glo®	Microscopic Evaluation
acemetacin	0	11.1
benzalkonium chloride	94.9	100
cefpodoxime proxetil	0	0
clofazimine	85.2	100
clofibric acid	0	0
docosanol	0	0
docusate sodium	0	0
ecamsule triethanolamine	0	0
eletriptan hydrobromide	0	0
etidronate disodium	0	0
flumazenil	0.5	0
lomerazine hydrochloride	78.6	100
methylbenzethonium chloride	84.5	100
nicardipine hydrochloride	76.8	88.9
perhexiline maleate	94.5	100
pipamperone	0	0
pipemidic acid	0	0
quinine ethyl carbonate	0	0
ritodrine hydrochloride	0	0
saccharin	0	0
spiramycin	0	0
sulpiride	0	0
tamoxifen citrate	85.4	100
teicoplanin	0	0
tetramizole hydrochloride	0	0

## Discussion

Concerns about increasing drug pressure due to the exclusive use of praziquantel underscore the need for new treatments [26]. The growing availability of new technologies, partnerships and open source drug discovery present great opportunities to screen more compounds for their antischistosomal activities, but to take advantage of them, effective, inexpensive and practical *in vitro* methods are required.

The aim of this research was to study a plethora of fluorescent or luminescent viability and cytotoxicity markers with various modes of action. In doing so, our hope was to develop a simple, novel and automated high-throughput *in vitro* drug sensitivity assay, as well as to elucidate types of colorimetric markers that are suitable for larval helminth screens.

For most of the tested markers, with the exception of LIVE/DEAD®, the signals tended to correlate well with an increased concentration of NTS (live NTS for viability markers and dead NTS for cytotoxicity markers). Moreover, the signals were usually differential between live and dead NTS for markers that reacted with NTS components that could be found in the medium, excluding CytoTox-ONE™ for which the difference was not at all significant. An issue for the markers that relied on staining NTS themselves was that signals were usually far too low to differentiate between live and dead NTS. Indeed, for markers that stained DNA of membrane-damaged NTS, only complete cell lysis would provide sufficiently high fluorescence signals. Since no drug completely lyses NTS, markers with this limitation could not be considered for further testing. In the case of LIVE/DEAD®, we attempted to increase the signal with a higher concentration of NTS and a smaller surface area to scan (using a 384-well plate), which was sufficient to render the signals differential between live and dead NTS (Additional file 4: Figure S2). However, the number of NTS required (1000 NTS) to achieve this and to negate the background noise is far too high to make for a realistically higher-throughput assay.

The largest issue that prevented markers from proceeding to further tests was that the background signals were far too high, meaning the S/B ratio could not exceed 3:1, our minimum standard. Issues with background signals were briefly explored in separate studies with culture medium (Additional file 5: Figure S3). The medium type and the presence of phenol red in the medium did tend to affect the signal, but this depended on the fluorescence marker used. The most notable impact on the signal, however, resulted from presence of iFCS in the medium, where removing it altogether would have the greatest impact on reducing background fluorescence. Nonetheless, previous studies have demonstrated that this is not a realistic option if NTS are to remain viable throughout the duration of a 24 or 72 hour drug assay [27].

Despite the above-described obstacles, resazurin, CellTiter-Glo® and Vybrant® could be tested in a drug-response assay. While a read-out with resazurin did not correlate at all with microscopic findings (signals were not differential enough to produce a curve), IC_50_ values could be generated using CellTiter-Glo® and Vybrant® that were close to the microscopic values for mefloquine. However, assays with Vybrant® could not produce a curve for oxamniquine. No marker gave signals that could be used to generate an IC_50_ value for praziquantel.

In the case of oxamniquine, the not entirely reproducible IC_50_ values are not altogether surprising since it is not highly active *in vitro* and even microscopic evaluations yield highly variable IC_50_ values. In contrast, microscopic evaluations for praziquantel usually do result in a typical dose–response curve. The lack of a dose–response relationship for praziquantel on NTS was observed also in previous studies using fluorescence/luminescent markers [9, 11, 18], and also when isothermal microcalorimetry was used [14]. It has been suggested that this is because praziquantel does not induce NTS death, which is true, but it does very severely damage them and reduce their motility, which should be reflected by reduced signals in such markers. It is therefore conceivable that while praziquantel does induce extensive damage, it also results in high enzyme or ion channel activity, which interacts with the viability markers and produces high signals.

Our comprehensive overview and studies are placed in context of previous studies with fluorescence and luminescence based NTS viability assays. Because of the Alamar Blue® experiments conducted by Mansour *et al*., [11] and resazurin experiments with *S. haematobium* [23] that did not yield promising results at the level of drug assay applicability, we did not expect resazurin to perform well as a marker and this was corroborated in our study. Our results mirror those of Mansour *et al*. [11], where the signal-to-NTS concentration tests shows promising results (albeit only after 24 hours of incubation with the marker), but the resazurin marker failed to generate dose-dependent viability curves and to distinguish between live and dead NTS after 72 hours of drug exposure.

The fluorescein diacetate/ propidium iodide assay published by Peak *et al.* is novel in that a duplex assay allows for simultaneous assessment of viability and cytotoxicity for *S. mansoni* adults and NTS [16]. The LIVE/DEAD® viability/cytotoxicity kit assay tested in our study functions in much the same way. Although Peak and colleagues had better success in obtaining concordant signals from both dyes, the fundamental issue with such markers in the end is the high number of NTS required to assess viability in a drug sensitivity assay, which reduces their throughput. Indeed, Lalli *et al*. [18] showed in their study that CellTiter-Glo® provides a far more sensitive assessment of parasite viability and, considering that it measures ATP production (which is theoretically abundant) after all the NTS have been completely lysed by the reagent, this is not surprising. In our study we also showed that CellTiter-Glo® can be used to assess drug dose–response effects. Furthermore, a screen of a 25 compound subset of an FDA-approved library showed 100 % correspondence between microscopic evaluation and CellTiter-Glo® with regards to hit identification, though all the active drugs in this screen were completely schistocidal.

In contrast to the study by Lalli and colleagues [18], we did see that if one wants to include compounds that damage but may not necessarily kill the worm as hits, the assay becomes less sensitive (as was shown with, for example, praziquantel). This lack of sensitivity could be due to variability in NTS concentration- if a well contains 100 NTS +/− 20 NTS, this already presents +/−20 % deviation in NTS concentration and in corresponding signal. Large signal variability might, therefore, impede the measurement of fine gradients of reduced viability. This might be the reason for large amounts of NTS used in previous studies [16] or why Lalli and colleagues [18] used a multi-drop sorter to dispense a more specific number of worms within the assay. Thus for a sensitive marker-based assay, much care needs to be undertaken to reduce NTS number variability.

## Conclusion

In summary, by testing a myriad of colorimetric markers with diverse mechanisms of action, we conclude that due to large fluctuations in signals, likely due to low numbers of NTS that are sensitive to variation in NTS concentration and viability, and high background noise, it is difficult to develop a simple, cheap “just add” colorimetric marker-based drug assay for the larval stage of *S. mansoni.* Markers that stain NTS themselves require a very large number of worms, and markers that assess elements spilled into the medium may require either a very specific number of worms or the removal of the assay supernatant in order to yield high and uniform signals. We could, however, confirm that CellTiter-Glo® may be used as a pre-screening tool in determining live and dead NTS in single drug concentration and potentially in dose–response assays.

## Additional files

Additional file 1: Table S1. Marker-specific methods used in this study. (DOCX 17 kb) Additional file 2: Table S2. Markers identified from literature review. (DOCX 34 kb) Additional file 3: Figure S1. Microscopic verification of staining. In the LIVE/DEAD® kit, calcein stains live NTS green (A, B) and EthD-1 stains dead cells red (C,D) already at 45 minutes. CellTox Green stains lysed NTS (E, F) already at 4 hours. Both Hoechst 33258 (G, H) and DAPI (I,J) stain even mefloquine-killed NTS well. (PPTX 1849 kb) Additional file 4: Figure S2. Fluorescence signals for live (green) and dead (red) NTS from a scan for the calcein reagent (A) and EthD-1 reagent (B) of LIVE/DEAD®. A 384-well plate assay was used and measured at various time-points- the curves shown here are for the 4-hour time-point. The background here is already subtracted (G,H). (PPTX 172 kb) Additional file 5: Figure S3. Fluorescence generated from culture medium as measured by (A) Omnicathepsin, (B) DAPI and (C) Hoechst 33258. Since our standard culture medium could also contribute to high fluorescence, RPMI medium was also tested. Graphs presented here correspond to optimal marker concentrations and incubation times are indicated in the main text. (PPTX 79 kb)
