# Supplementary material for: Fluorescence/luminescence-based markers for the assessment of Schistosoma mansoni schistosomula drug assays
Source: Parasit Vectors. 2015 Dec 8;8:624. doi: 10.1186/s13071-015-1233-3 (PMC4672532; doi:10.1186/s13071-015-1233-3)
Supplement: Additional file 1: Table S1. — Marker-specific methods used in this study. (DOCX 17 kb) [file 13071_2015_1233_MOESM1_ESM.docx]

| Marker Type | Marker Assay | Substance used to kill NTS | Final Assay Volume (µl) | Assay Reading Time-points | Wavelength settings (Ex/Em) |
| --- | --- | --- | --- | --- | --- |
| **Viability Markers** | CellTiter-Glo® | Ethanol 10% or DMSO 25% | 200 – 300 | every 10 minutes up to 2 hours | Luminescence |
|  | Resazurin | Ethanol 10% or DMSO 25% | 200 | every 10 minutes for the 1^st^ hour then at 90 min, 2, 3, 4, 6 and 24 hours | 555 nm/ 585 nm |
| **Cytotoxicity Markers** | Vybrant® | Cell lyser | 250 | every 10 minutes up to 3 hours and at 18 hours | 536 nm/ 588 nm |
|  | CytoTox-ONE™ | Cell lyser | 300 | every 10 minutes up to 1 hour, then at 1.5, 2, 3, 4, 6 and 24 | 560 nm/ 590 nm |
|  | CellTox™ Green Cytotoxicity Assay | Cell lyser, Ethanol 10%, DMSO 25%, MFQ 10μM | 200 | 0.25, 0.50, 0.75, 1, 2, 3, 4, 6, 24, 48 and 72 hours | 485 nm/ 520 nm |
| **Multiplex Markers** | LIVE/DEAD® Viability/Cytotoxicity Kit | Ethanol 10% or DMSO 25% | 300 | every 15 minutes for up to 4 hours and at 6, 10 and 24 hours | Calcein:  485 nm/ 525 nm  EthD-1:  525 nm/ 645 nm |
|  | ApoTox-Glo™ | Ethanol 10% or DMSO 25% | 220 | every 15 minutes up to 4 hours then at 6 and 24 hours | AFC:  400 nm/ 505 nm  R110:  485 nm/ 52 0nm |
| **Experimental Markers** | OmniCathepsin™ | Ethanol 10% or DMSO 25% | 200 | 0.25, 0.50, 0.75, 1, 2, 3, 4, 6 and 24 hours | 365 nm/ 440 nm |
|  | FluoForte® Calcium Assay | Cell lyser, Ethanol 10%, DMSO 25%, MFQ 10 μM | 100 | 0.50, 1, 1.50, 2, 3, 4, 6 and 24 hours | 490 nm/ 525 nm |
|  | DAPI | Cell lyser, MFQ 10 μM | 250 | 0.25, 0.50, 0.75, 1, 24, 48 and 72 hours | 358 nm/ 461 nm |
|  | Hoechst 33258 | Cell lyser, MFQ 10 μM | 200 | 0.25, 0.50, 0.75, 1, 2, 3, 4, 6 and 24 hours | 365 nm/ 440 nm |
