# Supplementary material for: Fluorescence/luminescence-based markers for the assessment of Schistosoma mansoni schistosomula drug assays
Source: Parasit Vectors. 2015 Dec 8;8:624. doi: 10.1186/s13071-015-1233-3 (PMC4672532; doi:10.1186/s13071-015-1233-3)
Supplement: Additional file 2: Table S2. — Markers identified from literature review. (DOCX 34 kb) [file 13071_2015_1233_MOESM2_ESM.docx]

| Type of marker/dye | Marker/dye | Mode of action^1^ | HTS Suitable?^2^ | Comment/ Issues | Reference |
| --- | --- | --- | --- | --- | --- |
| **Viability** | **Alamar Blue®** | Resazurin dye is cleaved by live cell redox reactions | Yes | Differentiates between live and dead but not live and damaged NTS | [11] |
|  | Carboxyfluoresceine-Diacetate | Dye is cleaved by intracellular esterases | No | Differential staining not possible  Leakage into the medium | [20] |
|  | **CellTiter-Glo®** | Luciferin reagent is cleaved by ATP produced from live cells | Yes* | May require large tissue dispenser for exact NTS numbers | [26] |
|  | L-lactate assay kit | Probe reacts with L-lactate, secreted by NTS | Yes* | Additional steps required: not quite high-throughput | [17] |
|  | MTT | Tetrazolium dye converted by live cell NADPH-dependent oxidoreductase enzymes | No | Laborious, toxic to live NTS when tested in our lab | [28, 29] |
|  | Neutral Red 1% | Vital stain- viable cells incorporate dye into their lysosomes | No | Differential staining not possible | [19] |
| **Cytotoxicity** | Ethidium bromide | Intercalates with DNA, membrane exclusion dye | No | Differential staining unsatisfactory | [20] |
|  | Methylene blue | Stains nucleic acids | No | Uptake is slow  Differential staining unsatisfactory. Toxic | [30, 31] |
|  | Propidium Iodide | Membrane exclusion dye | No | Differential staining unsatisfactory | [20] |
|  | Toluidine Blue | Stains nucleic acids | No | Tedious process  Differential staining unsatisfactory | [30] |
|  | Trypan Blue | Membrane exclusion dye | No | Differential staining unsatisfactory. Toxic | [19, 30] |
|  | **Vybrant®** | Reacts with dead cell G6PD release | ? | Measures G6PD release form dying cells. Not yet tested with *S. mansoni* | - |
|  | **CytoTox-ONE™** | Reacts with dead cell lactase dehydrogenase release | ? | Measures LDH release from dying cells. Not yet tested with *S. mansoni* | - |
|  | **CellTox™ Green Cytotoxicity Assay** | Membrane exclusion dye, intercalates with cellular DNA once membrane is compromised | ? | Membrane-exclusion dye, intercalates with DNA.  Not yet tested in assay form with *S. mansoni* | - |
| **Viability/ Cytotoxicity**  **Multiplex Assay** | Fluorescein-Diacetate  /Propidium Iodide | Fluorescein diacetate reacts with live cell esterases; propidium iodide intercalates with cellular DNA once membrane is compromised | Probable* | Laborious since several rinsing steps required. Large amounts of NTS necessary. | [16, 32] |
|  | **LIVE/DEAD® Viability/Cytotoxicity Kit** | Calcein AM reacts with live cell esterases, EthD-1 intercalates with cellular DNA once membrane is compromised | ? | Not yet tested on *S. mansoni*. | - |
|  | **ApoTox-Glo™** | GF-AFC substrate reacts with live cell protease activity; bis-AAF-R110 reacts with dead cell proteases | ? | Not yet tested on *S. mansoni* | - |
|  | Acridine Orange +  Ethidium Bromide | Acridine orange stains acidic vacuoles and nucleic acids in living cells; ethidium bromide stains DNA once membrane is compromised | No | Indiscriminate staining described for *S. mansoni* | [19] |
| **Experimental** | Bromothymol Blue | Stain to define cell walls | No | Did not stain schistosomula | [30] |
|  | **Diamidinophenylindole (DAPI)** | Intercalates with DNA- membrane permeable | ? | Stained live and dead schistosomula differentially for microscopy | [20] |
|  | Congo Red | Histological stain | No | Background coloring | [30] |
|  | Eosin | Histological stain | No | Background coloring noted | [30] |
|  | **Hoechst 33258** | Intercalates with DNA- membrane permeable | ? | Good differential staining described for *S. mansoni* eggs | [19] |
|  | **OmniCathepsin™** | Reacts with cathepsins | ? | Cathepsins are highly upregulated in NTS. Not yet tested on *S. mansoni* as a viability marker | [33] |
|  | **FluoForte® Calcium Assay** | detects Ca^2+^ mobilization; *S. mansoni* tegument is rich in calcium ion channels | ? | Not yet tested on *S. mansoni* | [34] |

*^1^ Mode of action determined from cell-based experiments.*

*^2^ HTS suitable means that marker assay can be read by an automated plate reader and does not require rinsing steps.*

** Needs to be optimized.*

*Markers in bold were selected for our study.*
