# Supplementary figures and images for: Fluorescence/luminescence-based markers for the assessment of Schistosoma mansoni schistosomula drug assays
Source: Parasit Vectors. 2015 Dec 8;8:624. doi: 10.1186/s13071-015-1233-3 (PMC4672532; doi:10.1186/s13071-015-1233-3)

## Slide 1
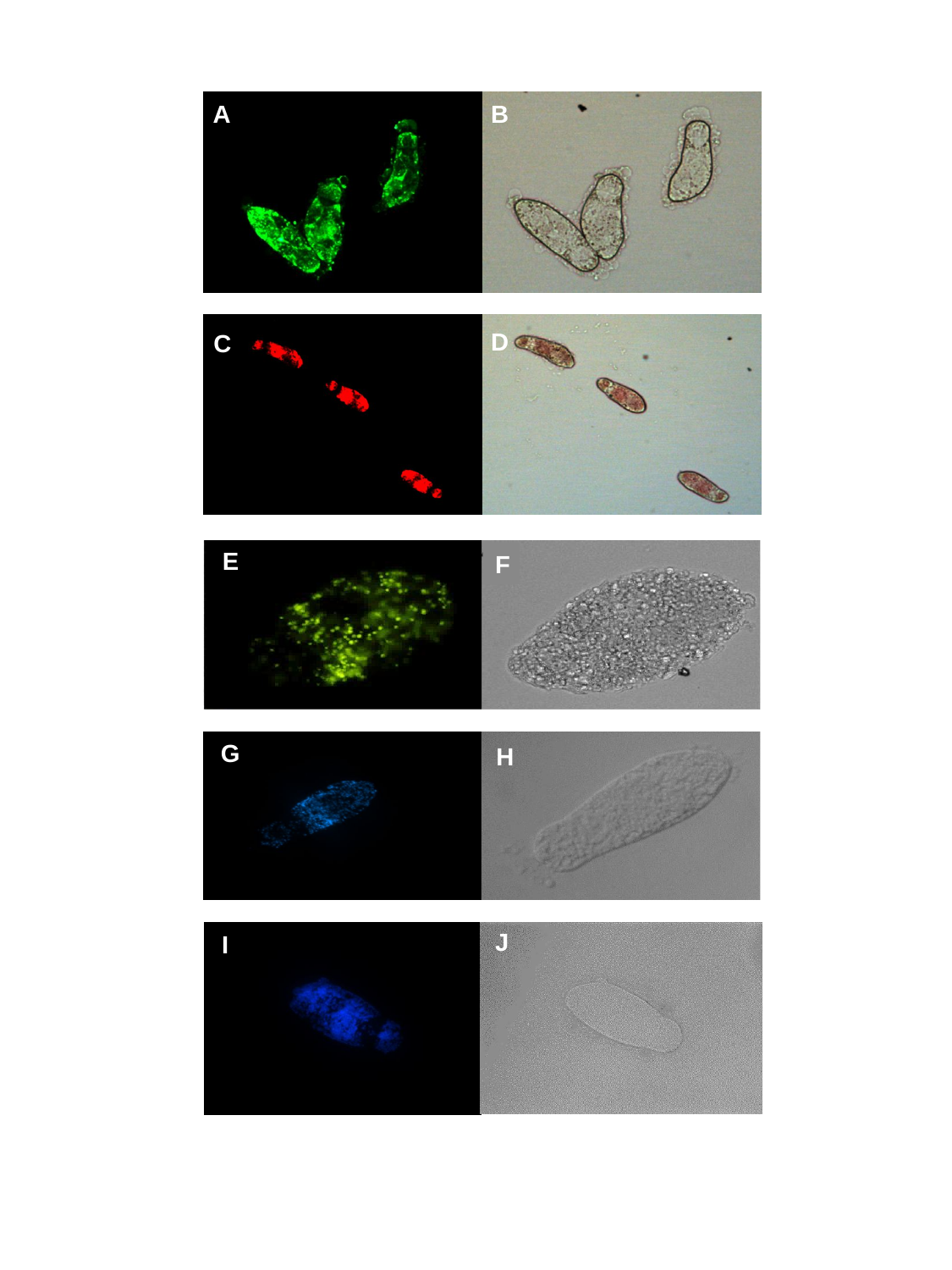

A
B
D
C
E
F
G
H
J
I
L
K

Supplement: Additional file 3: Figure S1. — Microscopic verification of staining. In the LIVE/DEAD® kit, calcein stains live NTS green (A, B) and EthD-1 stains dead cells red (C,D) already at 45 minutes. CellTox Green stains lysed NTS (E, F) already at 4 hours. Both Hoechst 33258 (G, H) and DAPI (I,J) stain even mefloquine-killed NTS well. (PPTX 1849 kb) [file 13071_2015_1233_MOESM3_ESM.pptx]

## Slide 1
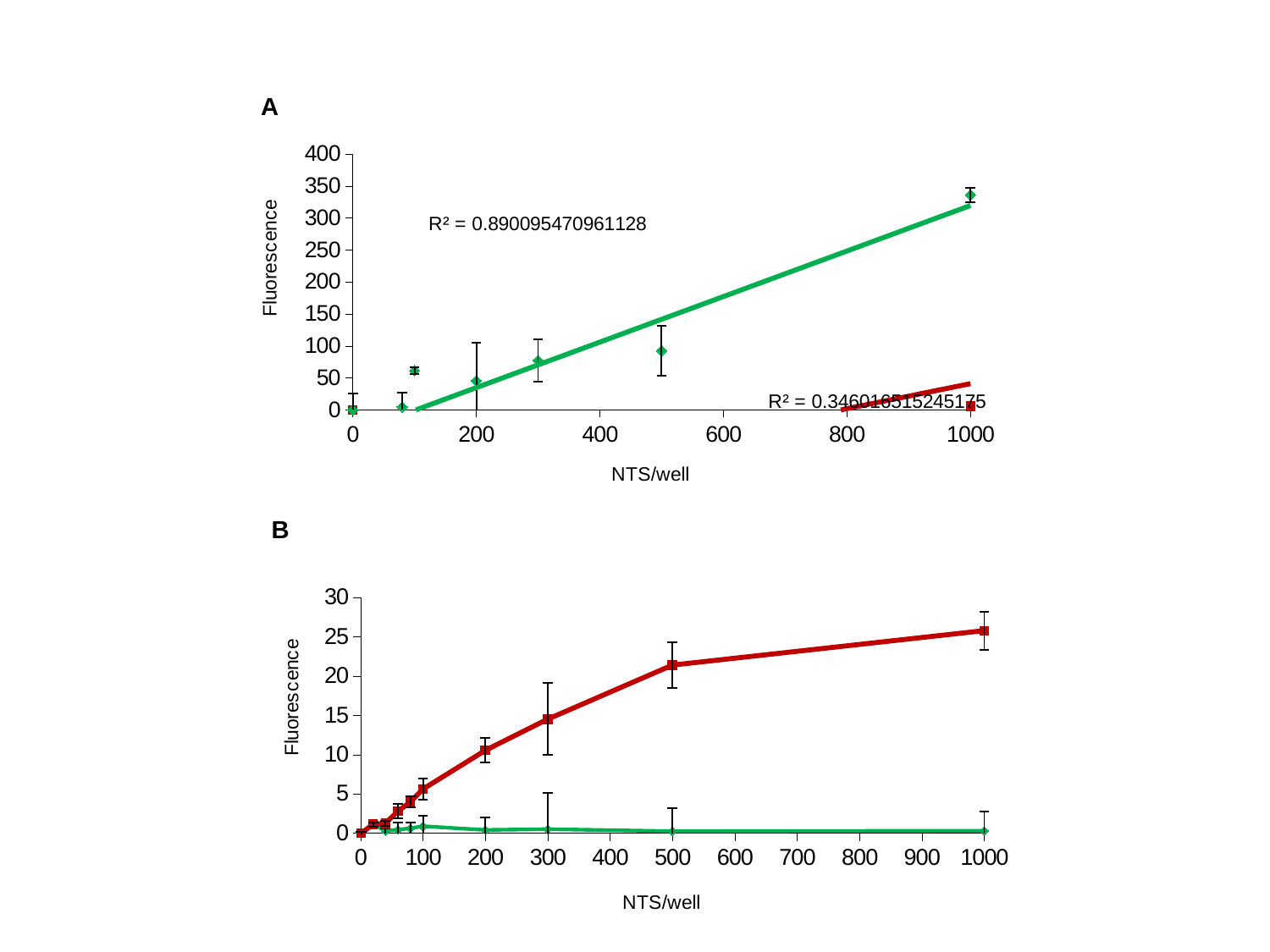

A
### Chart
| Category | | |
|---|---|---|B
### Chart
| Category | | |
|---|---|---|

Supplement: Additional file 4: Figure S2. — Fluorescence signals for live (green) and dead (red) NTS from a scan for the calcein reagent (A) and EthD-1 reagent (B) of LIVE/DEAD®. A 384-well plate assay was used and measured at various time-points- the curves shown here are for the 4-hour time-point. The background here is already subtracted (G,H). (PPTX 172 kb) [file 13071_2015_1233_MOESM4_ESM.pptx]
