# Supplementary material for: Fluorescence/luminescence-based markers for the assessment of Schistosoma mansoni schistosomula drug assays
Source: Parasit Vectors. 2015 Dec 8;8:624. doi: 10.1186/s13071-015-1233-3 (PMC4672532; doi:10.1186/s13071-015-1233-3)
Supplement: Additional file 5: Figure S3. — Fluorescence generated from culture medium as measured by (A) Omnicathepsin, (B) DAPI and (C) Hoechst 33258. Since our standard culture medium could also contribute to high fluorescence, RPMI medium was also tested. Graphs presented here correspond to optimal marker concentrations and incubation times are indicated in the main text. (PPTX 79 kb) [file 13071_2015_1233_MOESM5_ESM.pptx]

## Slide 1
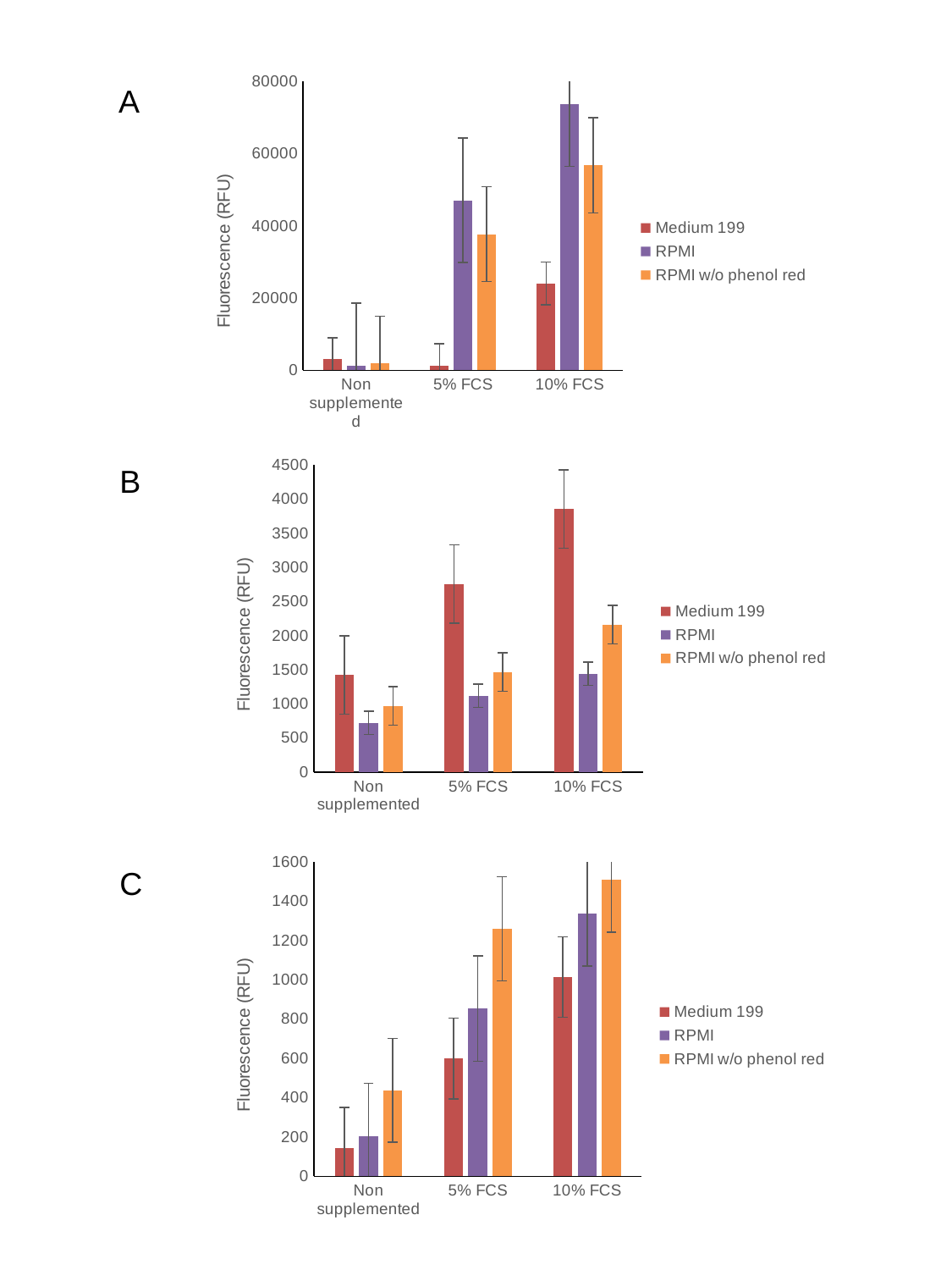

### Chart
| Category | | | |
|---|---|---|---|
| Non supplemented | 3059.2773333333334 | 1268.2436666666665 | 1854.1556666666668 |
| 5% FCS | 1321.8743333333332 | 47133.59533333333 | 37738.84766666667 |
| 10% FCS | 24045.070333333333 | 73827.607 | 56819.14566666667 |A
### Chart
| Category | | | |
|---|---|---|---|
| Non supplemented | 1424.0923333333333 | 719.8046666666665 | 971.7306666666667 |
| 5% FCS | 2756.057666666666 | 1122.2253333333333 | 1464.7743333333335 |
| 10% FCS | 3854.016333333333 | 1442.9796666666668 | 2160.9846666666667 |B
### Chart
| Category | | | |
|---|---|---|---|
| Non supplemented | 144.79566666666668 | 203.55933333333334 | 436.84400000000005 |
| 5% FCS | 599.2646666666666 | 853.505 | 1259.4889999999998 |
| 10% FCS | 1014.1303333333334 | 1338.832333333333 | 1507.2126666666666 |C
